# A Concise History of Asperger Syndrome: The Short Reign of a Troublesome Diagnosis

**DOI:** 10.3389/fpsyg.2015.02024

**Published:** 2016-01-25

**Authors:** J. B. Barahona-Corrêa, Carlos N. Filipe

**Affiliations:** ^1^Department of Psychiatry and Mental Health, Nova Medical School/Faculdade de Ciências Médicas - Universidade Nova de LisboaLisbon, Portugal; ^2^Neuropsychiatry Unit, Champalimaud Clinical Centre, Fundação ChampalimaudLisbon, Portugal; ^3^Centro de Apoio ao Desenvolvimento Infantil – CADINCascais, Portugal; ^4^Department of Psychiatry and Mental Health, Centro Hospitalar de Lisboa OcidentalLisbon, Portugal; ^5^Department of Physiology, Nova Medical School/Faculdade de Ciências Médicas - Universidade Nova de LisboaLisbon, Portugal

**Keywords:** Asperger Syndrome, autism spectrum disorders, DSM-5, psychopathology, nosology

## Abstract

First described in 1944 by Hans Asperger (1944), it was not before 1994 that Asperger Syndrome (AS) was included in the fourth edition of the Diagnostic and Statistical Manual of Mental Disorders, only to disappear in the Manual’s fifth edition in 2013. During its brief existence as a diagnostic entity, AS aroused immense interest and controversy. Similar to patients with autism, AS patients show deficits in social interaction, inappropriate communication skills, and interest restriction, but also display a rich variety of subtle clinical characteristics that for many distinguish AS from autism. However, difficulties operationalising diagnostic criteria and differentiating AS from autism ultimately led to its merging into the unifying category of Autistic Spectrum Disorders. Here we briefly review the short history of this fascinating condition.

## Introduction: Refrigerator Mothers and Fine-Boned Aristocrats

**FIGURE 1:**
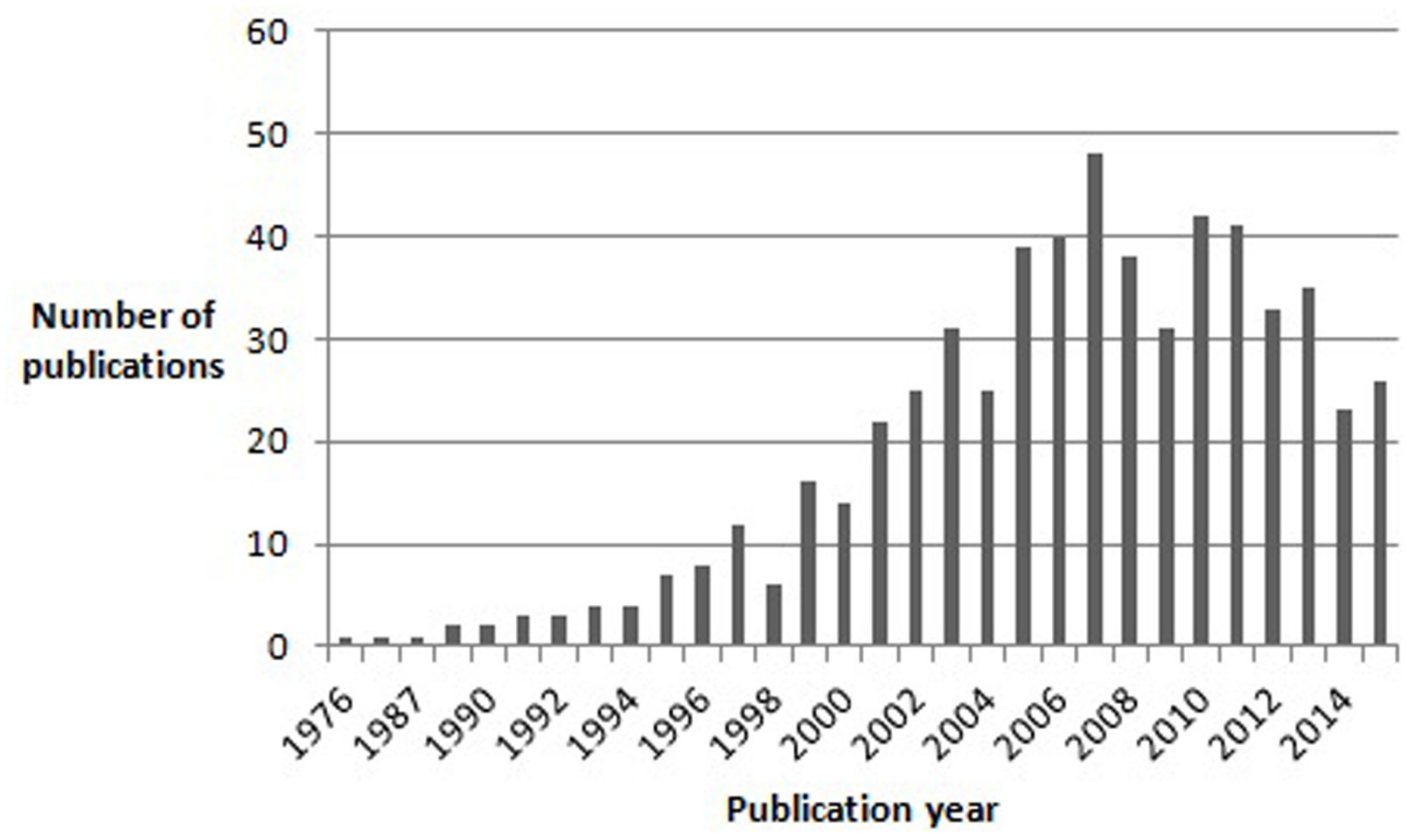
**Results of a pubmed search for articles containing the word “Asperger” in the title, published between 1976 and November 2015**.

The entry of autism and Asperger syndrome (AS) into the history of psychopathology was marked by extraordinary coincidences. Both disorders were first described by Kanner (1943) and Asperger (1944), respectively. Both were Austrian-born physicians and, though unaware of each other’s writings, both used the term “autistic” to describe a unique group of children who shared features of impaired social interaction and restricted, repetitive behaviors and interests. Both Kanner (1943) and Asperger (1944) borrowed the term “autistic” from Eugen Bleuler, who used it in his “Dementia Praecox or the Group of Schizophrenias” to describe extreme social withdrawal and self-centeredness in patients with schizophrenia. Moreover, both authors emphasized that the syndrome they were describing differed from infantile (e.g., De Sancti’s dementia praecocissima) and juvenile schizophrenia, namely by manifesting from birth and improving (in terms of social interaction) with growth, in contrast to the usual course of schizophrenia (Higier, 1923). Significantly, although Kanner (1943) initially considered language abnormalities (varying from sheer absence of language to atypical, socially ineffective use of well-developed language) to be a defining feature of his “Autistic Disturbances of Affective Contact,” he later hypothesized that they could be secondary to the two nuclear features of the disorder: “extreme self-isolation” and “obsessive insistence on sameness” (Irwin et al., 2011). Furthermore, Kanner (1943) also noted that many of his patients possessed “good cognitive potential.” Linguistic and cognitive ability would later sit in the eye of the storm unleashed by the appearance of AS as a discrete diagnostic entity and the relentless polemic that accompanied it. While Kanner’s syndrome eventually made its way to the third edition of the Diagnostic and Statistical Manual of Mental Disorders (DSM-III), Asperger’s work, published in German, remained virtually unknown to the international scientific community for almost half a century. Indeed, the first English translation of Asperger’s article “*Die Autistischen Psychopathen im Kindesalter”* first appeared in 1991 in Uta Frith’s textbook “autism and AS.” AS had already been described in 1981 by Lorna Wing, who first proposed the term to refer to a special subgroup of children who, according to Asperger’s original description, were characterized by: social isolation and lack of reciprocity in social interactions; normal or precocious language acquisition, with above-average linguistic skills but subtle abnormalities of verbal and non-verbal communication (e.g., atypical syntax, pedantic vocabulary and absent or stereotyped prosody); a narrow focus of interests, often restricted to unpragmatic and highly original themes; overachievement in specific cognitive domains; and motor clumsiness (Wing, 1981). Unlike Kanner (1943), Asperger (1944) did not attempt to define diagnostic criteria for the disorder he was describing. Moreover, Asperger greatly emphasized subtle positive features in his patients: they often had extremely original thought, they tended to cultivate abstract and intellectualized interests, often had, in Asperger’s own words, “a rare maturity of taste in art,” and even a peculiar, fascinating physical appearance, with “finely boned features,” of “almost aristocratic appearance” (Asperger, 1944). Asperger’s captivating descriptions of his subjects certainly played a decisive role in the history of the syndrome that bore his name, especially as they contrasted sharply with Kanner’s later recriminatory writings on “refrigerator-mothers” and the origin of autism (Irwin et al., 2011). Indeed, although Lorna Wing in her initial account of Asperger’s work clearly stated her belief that AS and Kanner’s autism were both part of an autistic spectrum, the idea of AS as an autonomous disorder, distinct from autism, quickly got hold of the opinion of many authors in the field, and certainly of the general public’s curiosity for autism and related disorders (Wing, 1994). It is important to note here that Asperger himself referred to Kanner’s paper, concluding that his subjects were clearly different from those described by Kanner (1943). The idea quickly made its way that Kanner’s autism and AS were different disorders, distinguished mainly by the fact that AS children had good cognitive and linguistic skills and a normal development in the first 2–3 years of life (Klin, 2003). Moreover, for many authors the impairment in social interaction differed qualitatively between AS and Kanner’s autism: while in the latter children seemed completely uninterested in others, AS children tried to relate with others but approached them in a dysfunctional and inconvenient way (Gillberg, 2002; Klin, 2003). Asperger himself contributed to this view that autism and AS subjects might be distinguished on the basis of cognitive ability and language development by emphasizing his patients’ high intelligence and their acquisition of grammatical speech before they could walk (Wing, 1994). The number of publications on AS grew exponentially in the years following Wing’s (1981) paper, and in 1994 AS was finally included in DSM – IV (**Figure 1**).

## Defining Asperger Syndrome: A Tower of Babel

**FIGURE 2:**
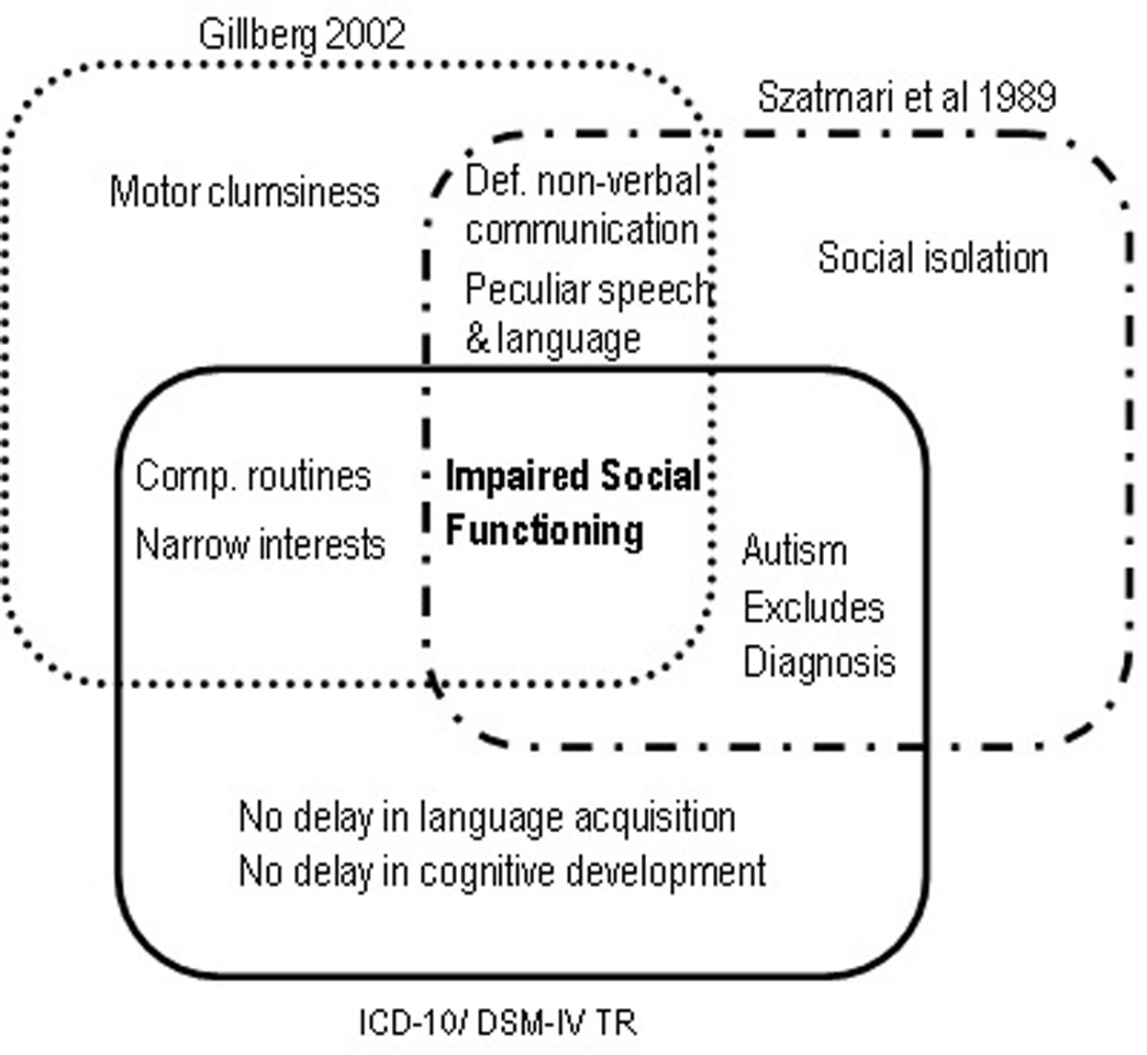
**Mandatory diagnostic criteria for Asperger Syndrome according to Szatmari et al. (1989), Gillberg (2002), ICD-10 and DSM-IV TR**.

DSM-IV broadened the diagnostic boundaries of Autism, conceived for the first time as a spectrum of disorders that included Autistic Disorder, AS and Pervasive Developmental Disorder Not Otherwise Specified (American Psychiatric Association [APA], 1994). Also new was the inclusion of Childhood Disintegrative Disorder (Heller Syndrome) and Rett Syndrome, both characterized by developmental regression with severe autistic features (Matson and Mahan, 2009). A diagnosis of Autism required only six symptoms (in contrast with the minimum of eight required in DSM-IIIR), including at least two social interaction deficits, two communication deficits, and one symptom of interest restriction/repetitive behavior. Functional impairment had to be obvious before age three. The newly created category of AS required at least two symptoms of social interaction deficits and one symptom of behavioral and interest restriction, normal cognitive, and linguistic development before age 3, and age-adequate adaptive functioning in areas other than social interaction. Onset before age three was not mandatory. Importantly, the subject should not meet diagnostic criteria for Autistic Disorder – in which case the latter diagnosis should be given precedence, implying a differential diagnosis between AS and autism without cognitive delay, also called high-functioning autism (HFA; Klin et al., 2005). Meanwhile, other sets of diagnostic criteria for AS had appeared (**Figure 2**). In 1988 Carina and Christopher Gillberg (2002) proposed six criteria based on Asperger’s original case-reports: socially impairing egocentricity, narrow interest patterns, compulsive routine adherence, peculiarities of speech and language, deficits in non-verbal communication, and motor clumsiness (Gillberg, 2002). Diagnosis required all six. There was no clause precluding a diagnosis of autism, and no mention of a minimum age limit or periods of normal development. 1 year later, Szatmari et al. (1989) proposed four mandatory criteria, comprising 22 symptoms: social isolation, impaired social functioning, deficits in non-verbal communication, and peculiarities of speech and language. As in DSM-IV, Autism was given diagnostic precedence over AS. Finally, WHO’s 1993 International Classification of Diseases and Disorders (ICD-10) also suggested diagnostic criteria for AS, essentially similar to DSM-IV’s (World Health Organization [WHO], 1992). Although not exactly contradictory, these several diagnostic schemes nevertheless produced a bewildering semiologic cacophony. Gillberg’s criteria are too restrictive, and the only mentioning clumsiness as a mandatory symptom. Szatmari’s criteria do not include interest restriction – a major criterion in the remaining diagnostic systems. DSM-IV and ICD-10 do not require abnormal non-verbal communication, mandatory in Gillberg’s and Szatmari’s sets. However, the most problematic clause, present in ICD-10, DSM-IV and Szatmari’s criteria, was the exclusion of a diagnosis of AS if criteria for autism were met.

## The End in the Beginning

The consecration of AS as a distinct diagnosis was surrounded by controversy from the outset. Contradictions in the syndrome’s definition soon became evident that would ultimately doom AS to extinction in DSM-5. The main problem was the precedence given to a diagnosis of autism. It soon became clear that most patients with significant impairments in social interaction and restriction of interests and activities also fulfill criteria for autistic disorder, thus precluding a diagnosis of AS. The requirement of normal cognitive and linguistic development failed to rescue a diagnosis of AS for the simple reason that cognitive and linguistic delay are not mandatory for diagnosing autistic disorder (Mayes et al., 2001; Happé, 2011). As Miller and Ozonoff (1997) demonstrated, even Asperger’s own initial cases would fail to qualify for a DSM-IV diagnosis of AS. Moreover, it is often difficult to establish retrospectively if a patient had normal language before the age of three, and full-scale IQ is seldom a useful measure in AS, given the typically heterogeneous IQ profile (Gillberg, 2002; Spek et al., 2008). Consequences of this conundrum soon became visible in research. Researchers used AS and HFA as interchangeable diagnoses, modified DSM or ICD criteria, or used original, investigator-specific criteria, compromising comparability across studies (Klin et al., 2005). Gradually, two positions regarding AS emerged in the field: (1) diagnosing AS using DSM-IV criteria is impossible because AS does not exist and is indistinguishable from HFA; (2) DSM-IV’s definition of AS is over-restrictive and additionally fails to discriminate AS from HFA (Szatmari, 2000; Mayes et al., 2001; Klin et al., 2005). Klin further argued that DSM-IV focuses excessively on superficial similarities between AS and HFA, ignoring AS’s unique features: presence of social motivation with awkward, one-sided social approaches, normal or precocious language with pragmatic deficits and one-sided verbosity, pretend play of unusual content, and circumscribed interests with inordinate gathering of information (Klin et al., 2005; Baron-Cohen and Klin, 2006). Importantly (albeit inconsequently), Klin proposed a reversal of the precedence rule: in the presence of criteria for both HFA and AS, As should be diagnosed.

## Is as Different from HFA?

Eventually, the controversy gradually converged onto knowing if AS and HFA can be distinguished qualitatively (suggesting different etiological and neurobiological mechanisms for each syndrome), or if they merely differ quantitatively and should therefore be regarded as variants of a single disorder (Macintosh and Dissanayake, 2004). Most studies used cross-sectional comparisons between subjects with either diagnosis to answer this question. Clinical differences between AS and HFA proved subtle at best. AS subjects have earlier language development, more appropriate intonation and pitch, and more pedantic speech and idiosyncratic vocabulary, while HFA subjects show more echolalia, pronoun reversal, and neologisms (Eisenmajer et al., 1998; Gilchrist et al., 2001; Macintosh and Dissanayake, 2004). AS children also display more imitative social play, attention and help-seeking, and reciprocal social interactions than HFA children (Prior et al., 1998; Macintosh and Dissanayake, 2006). Yet, these superior linguistic and social skills of AS children do not translate into superior ability to make friends or engage in reciprocal conversation. By adolescence, differences are no longer obvious, although AS subjects still show more sophisticated vocabulary and greater desire for friendship (Eisenmajer et al., 1998; Szatmari, 2000; Gilchrist et al., 2001; Wing et al., 2011). Cognitively, and as a group, AS subjects typically show a combination of superior verbal performance and visual-spatial, perceptual, and motor deficits (non-verbal learning disability profile), while the opposite profile characterizes HFA (Gillberg, 2002; Chiang et al., 2014). However, individual variability is huge, and it is difficult to control for the biasing effects of a differential diagnosis based on differences in language development (Klin et al., 2005). Studies that looked at theory of mind performance found mainly quantitative differences, with AS subjects scoring intermediately between HFA and healthy controls (Prior et al., 1998; Macintosh and Dissanayake, 2004).

Although many authors consider clumsiness as typical of AS, studies on motor control and gait have only found subtle differences in comparisons with HFA (Macintosh and Dissanayake, 2004; Rinehart et al., 2006). Again, differences decrease with age (Iwanaga et al., 2000). Finally, AS subjects show more intense preoccupations and circumscribed interests, while individuals with HFA have poorer imaginative play and more stereotyped behaviors, such as body rocking (Macintosh and Dissanayake, 2004; South et al., 2005). In terms of global functioning, AS subjects fare significantly better academically, but not in terms of employment or independent living (Tantam, 1994; Howlin, 2003).

An obvious approach to the question of whether or not AS and HFA are distinct entities is to look for biological differences between them. Despite the accumulated evidence on neurophysiological abnormalities in autism spectrum disorders (ASD) as a group, few data are available on possible differences between AS and HFA, apart from subtle differences in EEG connectivity patterns and left-hemisphere intra-cortical inhibition (abnormally decreased in HFA but not in AS; Duffy et al., 2013; Enticott et al., 2013; Luckhardt et al., 2014). Genetic studies have likewise produced little support for a discrimination between AS and HFA, although this must be tempered by growing evidence of a common genetic susceptibility shared by neurodevelopmental disorders in general, rather than a specific genetic etiology for each disorder (Lichtenstein et al., 2010).

Structural MRI studies comparing AS and HFA have produced contradictory results, with two recent meta-analyses and a recent systematic review reaching three different conclusions (Via et al., 2011; Yu et al., 2011; Pina-Camacho et al., 2013). The most consistent positive findings come from studies that differentiated AS from HFA based on language acquisition history: compared to AS and typical controls, HFA subjects have lower gray matter and white matter volumes, increased gyrification, and abnormal cortical folding in inferior frontal areas (including the pars opercularis; Nordahl et al., 2007; McAlonan et al., 2008, 2009; Jou et al., 2010); increased gray matter in supramarginal, superior temporal and inferior parietal gyri bilaterally (McAlonan et al., 2008; Jou et al., 2010; Toal et al., 2010), and decreased volume of the cerebellar vermis and posterolateral lobule (Scott et al., 2009; Hodge et al., 2010). However, these qualitative neuroanatomical differences are contradicted by studies that only found quantitative differences, with AS intermediately positioned between HFA and typical subjects (Lotspeich et al., 2004; Haznedar et al., 2006).

On the whole, data on the distinctiveness between AS and HFA remain difficult to interpret. Many differences are quantitative rather than qualitative, and are distinctly more pronounced at younger ages, although the same might be said of many differences that discriminate HFA from typically developing subjects (Luckhardt et al., 2014). Moreover, research has been systematically plagued by difficulties ensuring independence between selection criteria and outcome measures (Macintosh and Dissanayake, 2004). Diagnostic and assessment methodologies vary wildly across studies, with the direction of findings influenced by the inclusiveness of diagnostic criteria for AS, notably by whether language acquisition delay was used as a criterion for diagnosing HFA (Via et al., 2011). Importantly, most studies are cross-sectional, missing potential differences between developmental trajectories in AS and HFA (Pina-Camacho et al., 2013). Indeed there is increasing evidence that AS and HFA correspond to distinct developmental trajectories (McAlonan et al., 2008; Lotspeich et al., 2004), with HFA marked by a delay or failure in the transition from right to left hemisphere dominance, occurring at around age 3 in typically developing children (Szatmari, 2000; Rinehart et al., 2002). Ultimately, the insuperable fragility of AS as a diagnostic entity resides in the lack of a biological marker, and in a phenotype that many see as insufficiently different from that of other related disorders.

## A Foretold Resurrection?

Notwithstanding some clinical and biological differences between AS and HFA, the DSM-5 Neurodevelopmental Disorders Workgroup finally decided that there is insufficient evidence to support a meaningful distinction between them (Happé, 2011). DSM-5 therefore merged AS into a unitary category of autism spectrum disorders, characterized by a mandatory dyad of impaired social interaction and communication, and restricted, repetitive behaviors and interests (in contrast with the previously prevailing symptomatic triad; American Psychiatric Association [APA], 2013). All three symptoms of social interaction and communication deficits are required for a diagnosis. For the behavior and interest restriction criterion a polythetic definition was retained, albeit increasing the minimum of necessary symptoms from one to two, from a total of four (McPartland et al., 2012). Finally, a universal onset clause requires that symptoms be present from early childhood. Again, this profoundly modified definition of autism was met with criticism from the minute the first draft became known. Many patients and families, as well as adepts of the neurodiversity movement, were shocked that such an identity-defining diagnosis as AS ceased to exist overnight. Several specialists in the field felt that the decision was precipitate, that it ignored evidence supporting AS as a valid clinical and biological entity, and that DSM-5 fails to acknowledge the unique clinical features of those formerly diagnosed with AS (Wing et al., 2011; Spillers et al., 2014). Moreover, there are concerns that the new definition of ASD is too restrictive and will exclude many patients with AS from access to specialized treatment (Frazier et al., 2012; McPartland et al., 2012; Mayes et al., 2013). In fact, field trials showed that DSM-5 ASD has improved specificity at the cost of excluding more cognitively able individuals, including up to 75% of those previously diagnosed with AS (Frazier et al., 2012; Huerta et al., 2012; McPartland et al., 2012; Mayes et al., 2013). Concerns were further fueled by the inclusion of a new diagnosis of Social Communication Disorder in DSM-5, as this was felt by many to imply that higher functioning AS subjects would now migrate from the autistic spectrum to this new residual, consolation-prize category (Huerta et al., 2012). Others feel that the term AS should have continued to be mentioned in the manual as an admissible label for a particular group of patients within ASD, offering a clinical description of the syndrome but no diagnostic criteria (Wing et al., 2011). This would allow AS patients who regard the terms autism as unacceptably stigmatizing to keep their former diagnostic label. Indeed there is evidence that patients, families, education professionals, and health professionals connote AS with positive features and associate Autism with strange behavior, learning disability and family dysfunction (Kite et al., 2013; Spillers et al., 2014). Still others remain unreconciled with DSM-5 and hopeful of the syndrome’s rebirth in future revisions of the manual (Tsai, 2013). Regardless of whether or not there will be a future for AS as a valid and meaningful clinical construct, its short existence had the undeniable merit of boosting the public’s fascination with autism (Happé, 2011).

## Conflict of Interest Statement

The authors declare that the research was conducted in the absence of any commercial or financial relationships that could be construed as a potential conflict of interest. The reviewer João Gama Marques and handling Editor Diogo Telles-Correia declared their shared affiliation, and the handling Editor states that the process nevertheless met the standards of a fair and objective review.
